# At what level is the gap transfer illusion illusory?

**DOI:** 10.1177/20416695231194203

**Published:** 2023-08-21

**Authors:** Yoshitaka Nakajima, Gerard Bastiaan Remijn

**Affiliations:** 12923 Kyushu University, Japan

**Keywords:** audition, perceptual organization, illusory continuity, potential masking, Auditory Grammar

## Abstract

The gap transfer illusion is an auditory phenomenon in which a temporal gap in a longer glide transfers perceptually to a crossing shorter glide, making the longer glide illusorily continuous. This continuity is often considered a variation of classic illusory auditory continuity attributed to auditory peripheral activity, but a new view is given here supported by a series of sound demonstrations indicating that this illusory continuity is purely caused by a higher mechanism of perceptual organization.

If a 5000-ms descending glide and a 500-ms ascending glide cross one another (Sound 1-1 in Movie 1), they are typically perceived as two continuous glides in opposite directions. If a 100-ms gap is cut out in the shorter glide (Sound 1-2), two successive short tones are perceived, separated by a gap. However, if a gap is cut out in the longer glide (Sound 1-3), it is still perceived as continuous, leaving the gap in the shorter glide (Nakajima et al., 2022)—the *gap transfer illusion* (Nakajima et al., 2000). Sound 2 in Movie 2 is another version of this illusion.

Illusory continuity as in the longer glide of Sound 1-3 or Sound 2 tends to be considered a variation of classic *illusory auditory continuity* (Miller & Licklider, 1950; Warren, 2008). Simply stated, a physically continuous signal and the same signal made discontinuous with a gap or gaps provide acoustic cues that enable the auditory system to discriminate between them. Onsets, offsets, and silent parts covering a wide frequency range can be cues of discontinuity, while the absence of such cues can indicate continuity (Bregman, 1990; Nakajima et al., 2014). Strong sound(s) replacing the gap(s) mask the discontinuity cues, so that continuity is induced at the peripheral level. Neurophysiological correlates of such cues can be observed as single-neuron responses in A1 (Petkov et al., 2007). If two successive glides are on a single frequency trajectory and with a strong noise to fill a short gap between them, they tend to be perceived as a single continuous glide (Ciocca & Bregman, 1987).

In Sound 1-3, however, the discontinuity cues are not masked completely, and the presence of the gap makes the percept clearly different. Sound 1-1 (with a continuous longer glide) and Sound 1-3 (with a longer glide with a gap) are heard differently. The gap in Sound 1-3 is preserved perceptually, but in the shorter glide. Nakajima et al.'s (2014) *Auditory Grammar* explains this as follows (see also Nakajima et al., 2022; Remijn & Nakajima, 2005). The onset of the shorter glide and the offset just before the gap of the longer glide are mutually close in time, and there is enough sound energy to act as a filling between them, resulting in an illusory tone made of an onset, a filling, and an offset. The onset just after the gap of the longer glide and the offset of the shorter glide are also connected to construct another tone. The offset and the onset to begin and end the gap have been interpreted, and the silent part of the longer glide has been filled with a portion of the shorter glide. Thus, there remains no reason for the longer glide to be interpreted as discontinuous.

Sound 3-1 (Movie 3) is a new stimulus combining parts of Sound 2 and Sound 1-2, in which an ascending glide contains a gap. Here, a long, continuous ascending–descending tone in a lower zone, and two successive short tones with a gap in between in a higher zone are perceived. Both short tones should have been constructed through the connection of the closest onset and offset: the first tone illusorily and the second veridically. Combining different parts of Sound 2 and Sound 1-2, we obtain Sound 3-2, and two successive short tones are perceived in a similar way.

Now we get a critical view of the gap transfer illusion observing the fact that Sounds 1-2, 2, 3-1, and 3-2 *share the same physical time-frequency pattern* in their central neighborhoods of ∼500 ms: an ascending glide with a 100-ms gap and a continuously descending glide. A gap is always perceived *somewhere*: in the veridical (non-illusory) position in Sound 1-2, in a different glide in Sound 2, and in a succession of an illusory and a real tone in Sound 3-1 and Sound 3-2 (reversed). These demonstrations provide new evidence that continuity/discontinuity must have been determined by preceding and following information outside these central neighborhoods, within a fairly wide temporal window exceeding ∼200 ms. The difference between continuity and discontinuity cannot be explained via local peripheral activity resulting in classic auditory continuity. There should be an essential role of a higher perceptual mechanism that rearranges temporal pieces of auditory information into a global organization, covering a temporal range requiring a cognitive level, which often happens in vision and tactile sensation (e.g., apparent motion or facial expression perception).

## Supplemental Material

**Figure:** Video 1

**Figure:** Video 2

**Figure:** Video 3
